# Effects of intravenous human albumin, enteral cilostazol, and combination therapy on the reduction of delayed cerebral ischemia in patients with aneurysmal subarachnoid hemorrhage

**DOI:** 10.3389/fneur.2026.1889448

**Published:** 2026-07-16

**Authors:** Adnan I. Qureshi, Hassan Raza, Jonathan Beall, Byron Gajewski, Renee H. Martin, Jose I. Suarez

**Affiliations:** 1 Zeenat Qureshi Stroke Institute, Columbia, MO, United States; 2 Department of Neurology, University of Missouri, Columbia, MO, United States; 3 Department of Public Health Sciences, College of Medicine, Medical University of South Carolina, Charleston, SC, United States; 4 Department of Biostatistics and Data Science, University of Kansas Medical Center, Kansas City, KS, United States; 5 Division of Neurosciences Critical Care, Departments of Neurology, Anesthesiology and Critical Care Medicine, and Neurosurgery, Johns Hopkins University School of Medicine, Baltimore, MD, United States

**Keywords:** albumin, aneurysmal subarachnoid hemorrhage, Bayesian trial simulation, cerebral infarction, cilostazol, delayed cerebral ischemia, factorial component-effect decomposition

## Abstract

**Background:**

Delayed cerebral ischemia (DCI) and cerebral infarction remain major causes of disability and death after aneurysmal subarachnoid hemorrhage (aSAH). Enteral cilostazol and intravenous (IV) 25% human albumin have each been investigated as candidate therapies targeting potentially overlapping DCI mechanisms.

**Objective:**

This article aims to estimate the plausible effects of albumin, cilostazol, and combination therapy on reducing DCI after aSAH using Bayesian prior-predictive simulation and factorial component-effect decomposition.

**Methods:**

We defined four treatment classes: control, albumin-only, cilostazol-only, and combination therapy. The primary endpoint was the National Institute of Neurological Disorders and Stroke Common Data Elements-defined DCI (CDE-d-DCI) on computed tomography on days 10 to 14. A prior-predictive Monte Carlo model used a control DCI-risk prior centered on the Cilostazol Albumin Treatment in Subarachnoid Hemorrhage (CATS) protocol-planning value of 31%, a cilostazol prior anchored to randomized-trial meta-analysis estimates, and an albumin prior informed by the Albumin in Subarachnoid Hemorrhage (ALISAH) dose-escalation data with wider uncertainty because ALISAH was uncontrolled. No observed CATS outcomes were analyzed. External consistency, control-prior sensitivity, weaker albumin priors, correlated-prior structures, and factorial component effects were evaluated.

**Results:**

Under base-case priors, median absolute risk reductions (ARRs) versus control were 9.1 percentage points for albumin (90% simulation interval, −3.3 to 18.1; prior-predictive probability of ARR > 0, 89.8%), 18.1 percentage points for cilostazol (11.0 to 25.4; 99.9%), and 21.7 percentage points for combination therapy (12.4 to 29.9; 99.8%). Although combination therapy had the largest projected reduction versus control, the incremental benefit of adding albumin to cilostazol was small and uncertain (3.5 percentage points; −3.0 to 8.9), and the additive interaction was also uncertain (−5.5 percentage points; −11.9 to 2.8). Illustrative two-group sample sizes were 343, 76, and 49 participants per group for albumin, cilostazol, and combination therapy, respectively, versus control.

**Conclusion:**

These simulations provide planning-level projections and do not establish efficacy. The findings support continued randomized evaluation of cilostazol and albumin, with concurrent single-agent, combination, and control arms to estimate component effects and interaction effects.

## Introduction

The American Heart Association/American Stroke Association (AHA/ASA), the Neurocritical Care Society (NCS), and the European Stroke Organization (ESO) guidelines for the management of aneurysmal subarachnoid hemorrhage (aSAH) recognize that delayed cerebral ischemia (DCI) remains a major cause of death and disability in patients with aSAH (1–6). DCI contributes to poor outcomes through vasospasm-dependent and vasospasm-independent factors (7–10). Consensus definitions of DCI emphasize delayed neurological deterioration and/or cerebral infarction on imaging after excluding procedure-related infarction and other causes (10). Cerebral infarction is particularly important because it is more reproducible than clinical deterioration alone and is strongly associated with death or disability (9). National Institute of Neurological Disorders and Stroke Common Data Elements (NINDS CDE)-defined DCI (CDE-d-DCI) defines the endpoint as a new cerebral infarction on head computed tomography (CT) at approximately 2 weeks after randomization, with comparison of the days 10 to 14 or discharge CT to baseline and post-aneurysm-treatment imaging (11–13).

Oral nimodipine is the only agent shown to improve neurological outcomes but not cerebral vasospasm in patients with aSAH (AHA/ASA Class I recommendation, Level of Evidence A; NCS strong recommendation, moderate quality of evidence) (4, 5, 14). Other agents, such as endothelin-1 antagonists, magnesium sulfate, and statins, have failed to demonstrate consistent benefits in phase III clinical trials (15–21). Enteral cilostazol and intravenous (IV) 25% human albumin are two candidate agents evaluated for reducing DCI. Cilostazol is a phosphodiesterase-3 inhibitor with antiplatelet, vasodilatory, and potential anti-inflammatory effects (19, 22–24). Published randomized and non-randomized studies suggest that cilostazol reduces symptomatic vasospasm and DCI after aSAH, with an updated meta-analysis reporting a randomized-trial relative risk (RR) of 0.40 for new cerebral infarction (16, 25–27). IV 25% human albumin was evaluated in the Albumin in Subarachnoid Hemorrhage (ALISAH) dose-escalation trial, where high-dose albumin was feasible and associated with lower transcranial Doppler vasospasm, DCI, and cerebral infarction across dose tiers (28, 29). The evidence base is substantially stronger for cilostazol than for albumin (25, 28, 29).

The 2023 AHA/ASA guidelines have identified combination therapies as an important knowledge gap and future research area likely to offer the best chance of success in DCI prevention (4). This raises the possibility of combining both therapeutic agents in randomized controlled trials (RCTs). Before completion of the trial, investigators may need planning-level estimates of treatment effects, expected precision, the probability that a treatment class meets a prespecified decision threshold, and plausible mediation pathways. Aggregated historical and published data can support such a simulation, but they cannot alone identify causal mediation effects because the joint treatment-mediator-outcome distribution is generally unavailable. We present a scientific framework and illustrative estimates for a Bayesian prior-predictive simulation for future RCTs to quantify plausible effect ranges for IV albumin, enteral cilostazol, and combination therapy and to define the treatment-effect measures that should be evaluated once individual-level trial data are available. No observed outcomes were analyzed, and all estimates are conditional on the specified priors and model structure.

## Materials and methods

### Study context and treatment classes

For treatment-effect communication and component-effect planning, we created four clinically interpretable treatment classes: control, albumin-only, cilostazol-only, and albumin plus cilostazol combination therapy (Supplementary Table 1). Dose-specific analyses remain important secondary analyses because the class-level analyses collapse albumin durations and cilostazol doses and cannot identify an optimal regimen. However, class-level analyses provide stable estimates and directly address the clinical question of whether either agent, or the combination, reduces DCI in the Cilostazol Albumin Treatment in Subarachnoid Hemorrhage (CATS) trial.

### Primary outcome

The simulated primary outcome was CDE-d-DCI by days 10 to 14 or discharge CT, coded as a binary endpoint defined as new CT hypodensities adjudicated by a blinded imaging core laboratory that are not present on the 24- to 48-h post-aneurysm-treatment CT and are not attributed to surgical or endovascular treatment, ventriculostomy, intraparenchymal hematoma, hydrocephalus, or other non-DCI causes (10–13). Simulated treatment effects were reported on the absolute-risk scale as risk reduction per 100 treated patients because this scale is clinically interpretable and compatible with Phase II decision thresholds.

### External evidence and prior distributions

The control-risk prior was centered on the assumption that the control DCI risk is 31% (the CATS full protocol-planning value, based on preliminary published data) (9, 30, 31). In each Monte Carlo iteration, the control risk $p_{0}$ was sampled from Beta(31,69). The beta parameters were chosen so that the prior mean equaled the protocol-planning value of 0.31, while the effective sample size, defined as α+β , was 100. Thus, the prior can be interpreted as 31 effective historical CDE-d-DCI events and 69 effective non-events. This prior was used only to represent uncertainty in the expected control event rate and was not treated as observed trial data. This choice allows uncertainty around the baseline event rate while preserving the protocol-planning value. In the multiplicative prior-predictive model, $RR_{C}$ denotes the relative risk for cilostazol on the primary simulated outcome, CDE-defined delayed cerebral ischemia/cerebral infarction.

The prior for cilostazol was specified on the log-relative-risk scale using the randomized-trial meta-analysis estimate for new cerebral infarction (25). Specifically, $\log(RR_{C})$ was sampled from a normal distribution with a mean log(0.40) and standard deviation [log(0.67)−log(0.24)]/(2×1.96) , corresponding to a lognormal prior for $RR_{C}$ with a median of 0.40 and an approximate 95% prior interval of 0.24 to 0.67. The symptomatic vasospasm estimate, RR 0.46 with 95% CI 0.31 to 0.70, was not used as the primary outcome prior; it was retained only as supportive pathway evidence and, where applicable, as a separate mediator prior $RR_{C,M}$ . Because dose-specific evidence favoring 300 mg/day was mainly observational, the primary class-level simulation used the randomized-trial DCI estimate; dose–response simulations were discounted as observational dose comparisons (25). The prior albumin dosing was based on ALISAH, a multicenter, open-label, uncontrolled dose-escalation pilot trial showing the feasibility and tolerability of albumin doses up to 1.25 g/kg/day for 7 days and suggesting more favorable neurological outcomes at higher tiers (28). A subsequent ALISAH analysis reported lower frequencies of transcranial Doppler vasospasm, DCI, and cerebral infarction at higher albumin dosage tiers (29). Because these data were not definitive randomized efficacy evidence, albumin priors were assigned wider uncertainty intervals than cilostazol priors. The interaction of albumin plus cilostazol was centered near no interaction in the base-case analysis because direct human randomized evidence for the exact combination is limited. Sensitivity analyses allowed both modest synergy and antagonism. Prior distributions used for the illustrative Bayesian simulation are shown in Table 1. ALISAH enrolled 47 patients in sequential, open-label dose tiers without a concurrent randomized control group and was designed primarily to evaluate safety, tolerability, and maximum tolerated dose, rather than efficacy (28). The subsequent DCI and cerebral infarction observations were based on small tier-specific samples and incomplete follow-up imaging (29). Accordingly, the median albumin RR of 0.70 was a weakly favorable planning value, not an efficacy estimate calculated directly from ALISAH. In three observational studies, cilostazol 300 mg/day was associated with a delayed cerebral infarction rate of 4.1% versus 17.8% with 100 to 200 mg/day (RR, 0.27; 95% CI, 0.09 to 0.81). Still, this non-randomized comparison was not used to define dose-specific priors (25). ALISAH varied the daily albumin dose, whereas CATS varied the duration of the 1.25 g/kg/day regimen; ALISAH, therefore, cannot support 1-day versus 7-day duration-specific priors.

**Table 1:** Prior distributions used in the illustrative Bayesian simulation.

Parameter	Base-case prior	Rationale	Evidence strength
Control risk	p0 ~ Beta(31,69)	CATS protocol-planning mean based on preliminary published data (9, 30, 31)	Protocol-based; published support; sensitivity tested
Albumin RR (RR_A)	Median RR 0.70; 95% prior interval 0.40–1.20	Representative crude ALISAH (15%/31% = 0.48) discounted halfway toward the null on the log-RR scale; neutral-prior sensitivity tested	Very weak; subjective calibration
Cilostazol RR (RR_C)	Median RR 0.40; 95% prior interval 0.24–0.67	Anchored to randomized-trial meta-analysis for DCI	Moderate
Combination interaction RR (RR_AC)	Median RR 1.00; 95% prior interval 0.67–1.50	Centered on no multiplicative interaction; uncertainty allows synergy or antagonism	Very weak

Relative-risk priors were sampled from lognormal distributions parameterized by the stated median and 95% prior interval. ALISAH, Albumin in Subarachnoid Hemorrhage; CDE-d-DCI, Common Data Elements-defined delayed cerebral ischemia; DCI, delayed cerebral ischemia; RR, relative risk.

To provide a quantitative justification for the albumin planning prior, the ALISAH tier-specific DCI proportions of 20, 15, and 14% were compared descriptively with the 31% CATS planning control risk, corresponding to crude cross-study RRs of approximately 0.65, 0.48, and 0.45 (29). A representative RR of 0.48 was discounted halfway toward the null on the log-RR scale [exp{0.5 x log(0.48)} = 0.69] and rounded to 0.70. The 95% prior interval, 0.40 to 1.20, included no effect and plausible harm. This was a structured calibration rather than a formal multi-expert elicitation; the selection of the anchor and the degree of discounting remain subjective and may introduce optimism bias.

To assess this subjectivity, two weaker albumin priors were evaluated while holding the cilostazol and interaction priors fixed: a neutral prior (median RR 1.00; 95% prior interval, 0.67 to 1.50) and a diffuse-neutral prior (median RR 1.00; 95% prior interval, 0.50 to 2.00).

### Prior-predictive Monte Carlo simulation model

For each Monte Carlo iteration, the control risk p_0 was sampled from Beta(31,69). The relative-risk parameters were sampled independently from lognormal distributions, rather than treated as fixed values. For any relative-risk parameter with median m and central 95% prior interval [L,U], the simulation used RR ~ Lognormal(meanlog = log(m), sdlog^2), where sdlog = {log(U)−log(L)}/(2 × 1.96). Sampling, therefore, occurred on the log-relative-risk scale and was exponentiated back to the relative-risk scale.

In the base-case analysis, the explicit sampled distributions were RR_A ~ Lognormal(meanlog = log(0.70), sdlog = 0.280), RR_C ~ Lognormal(meanlog = log(0.40), sdlog = 0.262), and RR_AC ~ Lognormal(meanlog = log(1.00), sdlog = 0.206). These correspond to approximate central 95% prior intervals of 0.40 to 1.20 for albumin, 0.24 to 0.67 for cilostazol, and 0.67 to 1.50 for the albumin–cilostazol interaction, respectively.

In the base case, RR_A, RR_C, and RR_AC were sampled independently. This was a simplified assumption about prior uncertainty, not an assumption that therapies act through biologically independent mechanisms. Prior-dependence sensitivity analyses retained the same marginal priors. Still, they imposed a correlation coefficient rho = +0.50 or −0.50 between log(RR_A) and log(RR_C), with log(RR_AC) independent, and a positive joint-correlation structure in which all pairwise correlations equaled +0.50. These prespecified structures were stress tests and were not estimated from data.

Treatment-class risks were then generated under the multiplicative risk model:


$$\begin{array}{l} p\_A=p\_0\;x\;\mathrm{RR}\_A;p\_C=p\_0\;x\;\mathrm{RR}\_C;\\ p\_\mathrm{AC}=p\_0\;x\;\mathrm{RR}\_A\;x\;\mathrm{RR}\_C\;x\;\mathrm{RR}\_\mathrm{AC} \end{array}$$

where p_A denotes the albumin treatment risk, p_C denotes the cilostazol treatment risk, p_AC denotes the albumin–cilostazol combination treatment risk, RR_A denotes the relative risk for albumin, RR_C denotes the relative risk for cilostazol, and RR_AC denotes the relative-risk multiplier for the albumin–cilostazol interaction. The subscript C denotes cilostazol; the control risk is denoted p_0.

The conservative and optimistic sensitivity scenarios used the same log-normal parameterization. Conservative priors were RR_A ~ Lognormal(log(0.90), 0.256^2), RR_C ~ Lognormal(log(0.55), 0.211^2), and RR_AC ~ Lognormal(log(1.10), 0.193^2). Optimistic priors were RR_A ~ Lognormal(log(0.55), 0.307^2), RR_C ~ Lognormal(log(0.35), 0.280^2), and RR_AC ~ Lognormal(log(0.85), 0.253^2).

Treatment effects were summarized as absolute risk reduction (ARR = p_0−p_t), relative risk (RR = p_t/p_0), the prior-predictive probabilities that ARR exceeded 0, 5, and 10 percentage points, and the probability that each active-treatment class had the lowest CDE-d-DCI risk, where p_t denotes the treatment-class risk.

Three evidence-weighting scenarios were evaluated. The conservative scenario attenuated both component-effect priors and allowed modest antagonism. The base-case scenario used the priors described above and centered on the combination interaction with near-zero multiplicative interaction. The optimistic scenario used stronger priors for albumin and cilostazol, allowing modest potential synergy. Each scenario used 300,000 Monte Carlo iterations. These simulations estimate prior-predictive expectations, rather than posterior trial results. The reproducible base R script for these analyses is provided in Supplementary Appendix 2. Formal sensitivity analyses separately varied the control-prior mean and effective sample size while holding the base-case treatment-effect priors fixed. Because effective sample size is directly defined for the beta control-risk prior, effective-sample-size sensitivity was applied to that prior; uncertainty in the log-normal treatment-effect priors was evaluated through conservative, base-case, and optimistic scenarios. Additional sensitivity analyses used the two weaker albumin priors and the prespecified correlation structures described above.

### External-consistency, control-prior sensitivity, and sample-size calculations

Base-case projections were compared with published aggregate event rates from the cilostazol meta-analysis and ALISAH as an external consistency check, not as independent model validation, because these sources also informed the priors and did not uniformly use the CDE-d-DCI endpoint (25, 28, 29). Control-prior sensitivity analyses varied the control-risk mean and effective sample size. Albumin-prior and prior dependence analyses were performed as described above. Finally, illustrative two-group sample sizes were calculated from the simulated median risks to contextualize effect sizes; these calculations were not intended to replace full adaptive or factorial trial operating-characteristic simulations.

### Combination-effect estimand

Combination effects were evaluated on both the risk and absolute risk reduction scales. The primary class-level estimate compared combination therapy with control. A secondary additive interaction estimand assessed whether the combination exceeded the sum of its components on the absolute-risk-reduction scale:


$$\begin{array}{l} \text{Additive interaction}=\mathrm{ARR}\_\text{combination}-\\ \mathrm{ARR}\_\text{albumin}-\mathrm{ARR}\_\text{cilostazol} \end{array}$$

A positive value indicates a supra-additive risk reduction. A value near zero indicates additivity on the absolute-risk scale. A negative value indicates less-than-additive risk reduction on the absolute-risk scale. This statistic should be interpreted with caution because independent multiplicative risk reductions often produce a negative additive interaction, even when the combination has the lowest absolute risk.

### Factorial component-effect decomposition

Because IV albumin and enteral cilostazol are co-randomized treatment components, rather than temporally ordered variables, we used factorial component-effect decomposition, rather than natural mediation. A denotes assignment to IV albumin, C denotes assignment to enteral cilostazol, and p00, p10, p01, and p11 denote the potential DCI risks under control, albumin-only, cilostazol-only, and combination therapy, respectively. This approach separates the total effect of combination therapy from the individual component effects and their interactions, while avoiding the incorrect interpretation that one treatment mediates the other’s effect. The interaction term permits departure from multiplicative component effects and therefore does not require biologically independent mechanisms (3, 32–34).

The component absolute risk reductions were defined as ARR_A = p00−p10 for albumin alone and ARR_C = p00−p01 for cilostazol alone. The combination absolute risk reduction was ARR_AC = p00−p11. Incremental effects conditional on receipt of the other therapy were defined as Delta_A|C = p01−p11 for albumin added to cilostazol and Delta_C|A = p10−p11 for cilostazol added to albumin. Additive interaction was defined as ARR_AC−ARR_A−ARR_C; values greater than zero imply supra-additivity, values near zero imply additivity, and values less than zero imply sub-additivity on the absolute-risk scale.

In the base decomposition, the albumin and cilostazol RR priors were those used in the main prior-predictive simulation, with a weak-interaction prior centered on no multiplicative interaction. The prior for cilostazol was anchored to published meta-analytic evidence, whereas the albumin prior was intentionally wider because ALISAH was a dose-escalation pilot, rather than a definitive efficacy trial (25, 28, 29).

## Results

### Base-case prior-predictive treatment effects

In the base-case simulation, the median projected control DCI risk remained near the protocol assumption of 31% (9, 30, 31). Median projected DCI risks were 21.5% for albumin-only, 12.3% for cilostazol-only, and 8.6% for combination therapy. The corresponding median absolute risk reductions were 9.1, 18.1, and 21.7 percentage points, respectively (Table 2; Supplementary Figure 1). The prior-predictive probabilities that ARR exceeds 0 were 89.8% for albumin-only, 99.9% for cilostazol-only, and 99.8% for combination therapy. The prior-predictive probabilities that ARR exceeded 10 percentage points were 43.8, 96.9, and 97.5%, respectively. These probabilities summarize the specified priors and are not posterior probabilities of clinical effectiveness.

**Table 2:** Consolidated prior-predictive treatment-effects, sensitivity analyses, and interaction effects for National Institute of Neurological Disorders and Stroke Common Data Elements (NINDS CDE)-defined Delayed Cerebral Ischemia (DCI).

Estimate or scenario	Control	Albumin only	Cilostazol only	Combination	Interaction or probability details
Panel A: Base-case prior-predictive treatment-effect estimates					
Projected risk, median (90% interval)	30.9% (23.7 to 38.8)	21.5% (12.7 to 36.1)	12.3% (7.4 to 20.1)	8.6% (4.0 to 18.3)	—
ARR, median pp. (90% interval)	0.0 reference	9.1 (−3.3 to 18.1)	18.1 (11.0 to 25.4)	21.7 (12.4 to 29.9)	—
RR, median (90% interval)	1.00 reference	0.70 (0.44–1.11)	0.40 (0.26–0.62)	0.28 (0.14–0.57)	—
Pr ARR > 5 pp	Not applicable	73.5%	99.7%	99.4%	—
Pr ARR > 10 pp	Not applicable	43.8%	96.9%	97.5%	—
Pr ARR > 0	Not applicable	89.8%	99.9%	99.8%	—
Panel B: Sensitivity analysis for prior-predictive ARR					
Conservative ARR, pp	0.0 reference	3.0 (−11.5 to 13.0)	13.6 (6.0 to 20.7)	13.7 (−1.6 to 23.7)	—
Base-case ARR, pp	0.0 reference	9.1 (−3.3 to 18.1)	18.1 (11.0 to 25.4)	21.7 (12.4 to 29.9)	—
Optimistic ARR, pp	0.0 reference	13.6 (2.7 to 22.1)	19.7 (12.6 to 26.9)	25.3 (17.6 to 33.1)	—
Panel C: Combination interaction and probability of lowest-risk treatment class					
Conservative	Not applicable	Pr albumin lowest risk: 1.6%	Pr cilostazol lowest risk: 48.0%	Pr combination lowest risk: 50.3%	Additive interaction: −3.2 (−10.8 to 5.6); Pr supra-additive: 25.3%
Base case	Not applicable	Pr albumin lowest risk: 0.2%	Pr cilostazol lowest risk: 15.1%	Pr combination lowest risk: 84.7%	Additive interaction: −5.5 (−11.9 to 2.8); Pr supra-additive: 12.1%
Optimistic	Not applicable	Pr albumin lowest risk: 0.0%	Pr cilostazol lowest risk: 2.6%	Pr combination lowest risk: 97.3%	Additive interaction: −7.8 (−14.6 to 0.4); Pr supra-additive: 5.7%

Intervals are 5th–95th simulation percentiles, not frequentist confidence intervals. Conservative, base-case, and optimistic scenarios vary the component-effect and interaction priors. Panel C rankings compare the three active-treatment classes and exclude the control. ARR, absolute risk reduction; CDE-d-DCI, Common Data Elements-defined delayed cerebral ischemia; DCI, delayed cerebral ischemia; NINDS CDE, National Institute of Neurological Disorders and Stroke Common Data Elements; pp, percentage points; Pr, probability; RR, relative risk.

### External-consistency and control-prior sensitivity

The base-case cilostazol relative risk of 0.40 reproduced the randomized-trial meta-analytic anchor by design. The projected control and cilostazol risks of 30.9 and 12.3%, respectively, were directionally consistent with published rates of 26.6 and 10.1% pooled across randomized and observational studies (25). The projected albumin risk of 21.5% was descriptively comparable with ALISAH DCI frequencies of 14 to 20%, whereas cerebral infarction occurred in 25 to 45% of the small subset with follow-up CT imaging (29). These comparisons support consistency with the source of evidence but do not constitute independent validation. Varying the control-risk mean from 26.6 to 35.0% changed the magnitude of the ARRs but not the treatment ranking. Varying the control-prior effective sample size from 25 to 200 primarily widened or narrowed the simulation intervals, with little effect on median estimates (Supplementary Tables 2, 3).

### Illustrative sample-size implications

Using the base-case median projected risks, separate two-group calculations at 80% power and two-sided alpha = 0.05 required approximately 343 participants per group for albumin versus control, 76 per group for cilostazol versus control, and 49 per group for combination therapy versus control. With alpha = 0.0167 for three active-versus-control comparisons, the corresponding estimates were 457, 102, and 66 participants per group. Conservative prior assumptions substantially increased the required sample size for albumin, consistent with the greater uncertainty around the albumin prior (Supplementary Table 4).

### Scenario analyses

Projected ARRs remained larger for cilostazol and combination therapy than for albumin alone across conservative, base-case, and optimistic assumptions (Table 2; Supplementary Figure 2). Albumin-only estimates were more sensitive to prior strength, consistent with the weaker evidence base supporting the albumin prior.

### Albumin-prior and prior-dependence sensitivity

When the albumin prior was centered at RR 1.00, the median albumin ARR was 0.0 percentage points under both the neutral prior (90% simulation interval, −12.6 to 9.0; prior-predictive Pr[ARR > 0], 50.0%) and the diffuse-neutral prior (−24.7 to 13.8; 50.0%). The median combination ARR decreased from 21.7 percentage points in the base case to 18.0 percentage points in both settings. Cilostazol and combination therapy were each the lowest-risk active-treatment class in approximately 50% of simulations (Supplementary Table 5).

Correlated-prior analyses preserved the median combination ARR at approximately 21.5–21.8 percentage points but changed its uncertainty. Under positive joint correlation among all three log-relative-risk parameters, the 90% interval widened to 7.0–30.8 percentage points, and the probability that combination therapy was the lowest-risk active-treatment class decreased from 84.7 to 80.0%. The correlation between the component effects alone had little effect on ranking (Supplementary Table 6).

### Probability of the lowest-risk treatment class and combination interaction

Combination therapy had the highest probability of being the lowest-risk active-treatment class in both the base-case and optimistic scenarios. In the base case, combination therapy had the lowest DCI risk in 84.7% of simulations, compared with 15.1% for cilostazol-only and 0.2% for albumin-only. These are prior-predictive ranking probabilities; combination interaction estimates and ranking probabilities are summarized in Table 2.

### Factorial component-effect decomposition

Under the base-case weak-interaction prior, the median projected control DCI risk was 30.9% (90% simulation interval, 23.7 to 38.8%). The median projected DCI risks were 21.5% for albumin-only, 12.3% for cilostazol-only, and 8.6% for combination therapy, corresponding to median ARRs of 9.1, 18.1, and 21.7 percentage points, respectively (Table 3).

**Table 3:** Factorial component-effect decomposition for reduction of NINDS CDE-defined DCI.

Treatment condition	Median projected DCI risk	90% simulation interval	ARR versus control, median pp	Interpretation
Control	30.9%	23.7 to 38.8%	Reference	Reference DCI risk under no albumin and no cilostazol
Albumin only	21.5%	12.7 to 36.1%	9.1	Projected DCI risk with albumin but no cilostazol
Cilostazol only	12.3%	7.4 to 20.1%	18.1	Projected DCI risk with cilostazol but no albumin
Combination therapy	8.6%	4.0 to 18.3%	21.7	Projected DCI risk with both therapies

Values are medians and 90% simulation intervals from the base-case weak-interaction prior. The factorial decomposition does not imply biological independence; RR_AC permits departure from multiplicative component effects. ARR, absolute risk reduction; DCI, delayed cerebral ischemia; NINDS CDE, National Institute of Neurological Disorders and Stroke Common Data Elements; pp, percentage points.

Under the base-case priors, the median incremental ARR for albumin added to cilostazol was 3.5 percentage points (90% interval, −3.0 to 8.9), whereas the median incremental ARR for cilostazol added to albumin was 12.4 percentage points (90% interval, 5.5 to 22.8). The additive interaction was −5.5 percentage points (90% interval, −11.9 to 2.8), indicating sub-additivity on the absolute-risk scale, rather than clear evidence of supra-additive synergy.

## Discussion

Our analysis demonstrates how published aggregate evidence can be used to generate clinically interpretable treatment-effect projections before trial outcome data are available. These patterns reflect the relative strength of the external evidence: Cilostazol has RCT-based meta-analysis support for reducing DCI, whereas albumin is supported mainly by feasibility and dose–response pilot data from an uncontrolled dose-escalation study (25, 28, 29). The external-consistency comparisons should not be interpreted as independent validation because the same evidence sources informed the priors and those sources differ from those of the planned CATS endpoint and population. The simulation framework remains useful for Phase II planning because it translates uncertain prior evidence into ARRs, prior-predictive probabilities that ARR exceeds specified thresholds, treatment-ranking probabilities, and illustrative sample-size implications (1, 2, 35). The approach also separates three concepts that are often conflated: total treatment effect, additive interaction, and mechanistic mediation (3, 32–34). These simulations provide planning-level projections and do not establish efficacy. Under the specified base-case priors, cilostazol had the largest single-agent projected ARR, 18.1 percentage points versus control (90% simulation interval: 11.0 to 25.4), with a prior-predictive Pr[ARR > 0] of 99.9%. Albumin had a median projected ARR of 9.1 percentage points versus control and a Pr[ARR > 0] of 89.8%, although its uncertainty interval was wider (−3.3 to 18.1), reflecting the weaker external evidence base. Combination therapy had the largest projected ARR, 21.7 percentage points versus control (90% simulation interval: 12.4 to 29.9); however, the incremental albumin effect when added to cilostazol was modest and uncertain (3.5 percentage points; 90% simulation interval: −3.0 to 8.9), and the additive interaction estimate was also uncertain (−5.5 percentage points; 90% simulation interval: −11.9 to 2.8). Under neutral albumin priors, the median albumin ARR was 0.0 percentage points, and cilostazol and combination therapy were each the lowest-risk active-treatment class in approximately half of the simulations, indicating that the base-case combination ranking depends materially on the favorable albumin prior. A future factorial RCT should therefore retain concurrent arms for cilostazol alone, albumin alone, combination, and control so that component and interaction effects can be formally estimated within the same randomized population.

The biological rationale for evaluating combination therapy is also consistent with the original CATS scientific premise. Cilostazol may affect several mechanisms relevant to DCI, including vasodilation, inhibition of platelet aggregation, inhibition of smooth muscle proliferation, protection of vascular endothelial cells, suppression of inflammatory adhesion molecules, and reduction of spreading depolarization-related injury (19, 22–24, 36–44). Human albumin may act through partly overlapping and potentially complementary mechanisms, including improved microvascular perfusion through rheological effects, blood–brain barrier stabilization, neuroprotection through antioxidant and intracellular signaling pathways, reduction in cellular edema, and mitigation of post-ischemic thrombosis (28, 45–50). The purpose of the simulation was not to prove synergy, but to estimate how plausible component effects and uncertain interaction effects might translate into ARR. The choice of CDE-d-DCI as the simulated endpoint is important for interpreting these results. Angiographic or symptomatic vasospasm alone may not fully capture the vasospasm-dependent and vasospasm-independent pathways leading to poor outcomes after aSAH. Cerebral infarction is a more reproducible and clinically relevant endpoint because it can be assessed on imaging, can be adjudicated centrally, and is strongly associated with death or disability (9–13).

The base-case model sampled RR_A, RR_C, and RR_AC independently, but this statistical assumption concerns uncertainty in the parameter values, rather than biological pathway independence. The selected correlation structures had little effect on the median combination ARR but did affect its uncertainty, particularly when interaction uncertainty was correlated with the component effects. Other reasonable formulations include a multivariate prior with elicited covariance, robust mixture priors, meta-analytic predictive or commensurate/power priors, and hierarchical partial pooling across albumin-duration and cilostazol-dose cells (51, 52). Because the available aggregate data do not identify the covariance structure, these alternatives were not fitted in the present analysis.

Several limitations require emphasis. First, these simulations are not based on an RCT evaluating the combination and are intended as planning-level projections under specified priors rather than evidence of efficacy. Second, the external evidence may differ from the future CATS trial in terms of patient population, timing of treatment, aneurysm-treatment modality, imaging practices, standard-of-care cointerventions, and endpoint definitions. Third, the 31% control risk is a protocol-planning value based on preliminary published data, rather than a pooled estimate developed specifically for this simulation. However, sensitivity analyses varying the control-risk mean and effective sample size showed that the treatment ranking was unchanged. Fourth, although the albumin RR of 0.70 now has a quantitative rationale, selection of the ALISAH anchor and degree of discounting remain subjective; no formal multi-expert elicitation was performed, and optimism bias cannot be excluded. The neutral and diffuse-neutral albumin-prior analyses reduce but do not eliminate this concern. Fifth, independent prior sampling was a simplifying assumption. The selected correlation analyses showed that dependence primarily affected combination uncertainty, but other covariance structures could produce different results. Sixth, the class-level analysis collapses albumin duration and cilostazol-dose arms, which improves interpretability but can obscure dose–response or duration-specific heterogeneity and cannot identify an optimal regimen. Seventh, the illustrative sample-size calculations are simple two-group approximations and do not replace full adaptive or factorial trial operating-characteristic simulations. Eighth, the multiplicative risk model is convenient but not the only reasonable data-generating model. Finally, aggregate published data cannot identify natural direct and indirect effects, and future mediation analyses using CATS data will remain sensitive to mediator measurement, missingness, and unmeasured mediator–outcome confounding. These limitations reinforce that the current estimates are planning-level projections rather than evidence of definitive efficacy.

## Conclusion

A Bayesian prior-predictive simulation can provide useful planning-level estimates of the effects of IV human albumin, enteral cilostazol, and combination therapy on reducing DCI after aSAH, but it does not establish treatment efficacy. Under base-case assumptions, cilostazol and combination therapy had the largest projected DCI reductions; under neutral albumin priors, their ranking was approximately equal. The uncertainty around albumin and additive interactions supports retaining concurrent control, albumin-only, cilostazol-only, and combination arms in future randomized evaluations so that component and interaction effects can be estimated within the same trial population.

## Data Availability

The original contributions presented in the study are included in the article/Supplementary material, further inquiries can be directed to the corresponding author.
