# Supplementary material for: Effects of intravenous human albumin, enteral cilostazol, and combination therapy on the reduction of delayed cerebral ischemia in patients with aneurysmal subarachnoid hemorrhage
Source: Front Neurol. 2026 Jul 16;17:1889448. doi: 10.3389/fneur.2026.1889448 (PMC13421411; doi:10.3389/fneur.2026.1889448)
Supplement: Supplementary file 1 [file Supplementary_file_1.docx]

**Supplementary Material**

**Supplementary Appendix 1. Treatment classes and prior-predictive figures**

This appendix contains the supplementary tables and figures cited in the main manuscript.

**Supplementary Table 1.** Treatment classes for simulation analysis.

| **Class** | **CATS arms represented** | **Intervention definition** | **Primary contrast** |
| --- | --- | --- | --- |
| Control | Placebo/saline arm | Saline infusion plus placebo enteral tablets | Reference |
| Albumin only | Albumin arms without cilostazol | 25% human albumin 1.25 g/kg/day for 1 or 7 days | Albumin only vs control |
| Cilostazol only | Cilostazol arms without albumin | Enteral cilostazol 200 or 300 mg/day for 14 days | Cilostazol only vs control |
| Combination | Albumin plus cilostazol arms | Any albumin duration plus any cilostazol dose | Combination vs control and vs component therapies |

*Note. Dose-specific analyses should preserve the full 3 by 3 factorial structure. Class-level analyses are proposed for primary interpretability and to improve stability in planning simulations.*

*Abbreviations: CATS, Cilostazol Albumin Treatment in Subarachnoid Hemorrhage; CTZ, cilostazol; HA, human albumin.*

**Supplementary Table 2.** External consistency of prior-predictive projections with published aggregate evidence.

| **Evidence domain** | **Published aggregate data** | **Base-case model projection** | **Interpretation** |
| --- | --- | --- | --- |
| Control-risk context | New cerebral infarction: 26.6% across pooled control groups from randomized and observational studies (reference 25). | Control risk: 30.9% (90% interval, 23.7 to 38.8). | Broad contextual consistency; 31% is the CATS protocol-planning value based on values derived from preliminary published data (main manuscript references 9, 30, and 31). |
| Cilostazol | New cerebral infarction: 10.1% with cilostazol versus 26.6% control across randomized and observational studies; randomized-trial RR 0.40 (95% CI, 0.24 to 0.67) (reference 25). | Risk: 12.3% versus 30.9%; median RR 0.40 (90% interval, 0.26 to 0.62). | Consistency is expected because the randomized-trial RR informed the prior; this is not independent validation. |
| Albumin | ALISAH DCI: 20%, 15%, and 14% across dose tiers; cerebral infarction: 45%, 27%, and 25% among patients with follow-up CT (references 28 and 29). | Albumin risk: 21.5% (90% interval, 12.7 to 36.1). | Descriptive comparison only because ALISAH was uncontrolled and differed in dose tiers, imaging availability, and endpoint timing. |
| Combination therapy | No directly comparable randomized human data. | Combination risk: 8.6% (90% interval, 4.0 to 18.3). | External validation is not currently possible. |

*Note. These comparisons assess consistency with the evidence used to construct the priors and should not be interpreted as independent validation.*

*Abbreviations: CI, confidence interval; CT, computed tomography; DCI, delayed cerebral ischemia; RR, relative risk.*

**Supplementary Table 3.** Sensitivity to the control-risk prior mean and effective sample size.

| **Control-prior setting** | **Control risk, median (90% interval)** | **Albumin ARR, median pp (90% interval)** | **Cilostazol ARR, median pp (90% interval)** | **Combination ARR, median pp (90% interval)** |
| --- | --- | --- | --- | --- |
| **Panel A. Control-risk mean sensitivity** |  |  |  |  |
| Mean 26.6%; ESS 100 | 26.4% (19.7 to 34.1) | 7.7 (-2.9 to 15.7) | 15.5 (9.3 to 22.2) | 18.5 (10.4 to 26.2) |
| Mean 31.0%; ESS 100 | 30.9% (23.7 to 38.8) | 9.1 (-3.3 to 18.1) | 18.1 (11.0 to 25.4) | 21.7 (12.4 to 29.9) |
| Mean 35.0%; ESS 100 | 34.9% (27.4 to 43.0) | 10.3 (-3.9 to 20.3) | 20.6 (12.6 to 28.3) | 24.5 (14.1 to 33.3) |
| **Panel B. Effective-sample-size sensitivity at mean 31.0%** |  |  |  |  |
| Mean 31.0%; ESS 25 | 30.5% (17.0 to 46.8) | 8.5 (-3.3 to 19.9) | 17.7 (8.6 to 29.6) | 21.0 (9.8 to 35.0) |
| Mean 31.0%; ESS 50 | 30.8% (20.8 to 42.1) | 8.8 (-3.3 to 18.8) | 18.0 (10.1 to 27.0) | 21.4 (11.5 to 31.9) |
| Mean 31.0%; ESS 100 | 30.9% (23.7 to 38.8) | 9.1 (-3.3 to 18.1) | 18.1 (11.0 to 25.4) | 21.7 (12.4 to 29.9) |
| Mean 31.0%; ESS 200 | 30.9% (25.8 to 36.5) | 9.2 (-3.4 to 17.7) | 18.3 (11.5 to 24.3) | 21.9 (12.8 to 28.6) |

*Note. The base-case treatment-effect and interaction priors were held fixed. Changing the prior mean altered the absolute scale of benefit, whereas changing effective sample size primarily changed uncertainty. Intervals are 5th to 95th simulation percentiles.*

*Abbreviations: ARR, absolute risk reduction; ESS, effective sample size; pp, percentage points.*

**Supplementary Table 4.** Illustrative sample-size implications of the simulated median risks.

| **Scenario** | **Active-versus-control comparison** | **Control risk** | **Active risk** | **n per group, alpha 0.05** | **n per group, alpha 0.0167** |
| --- | --- | --- | --- | --- | --- |
| Base case | Albumin only | 30.9% | 21.5% | 343 | 457 |
| Base case | Cilostazol only | 30.9% | 12.3% | 76 | 102 |
| Base case | Combination | 30.9% | 8.6% | 49 | 66 |
| Conservative | Albumin only | 30.9% | 27.6% | 3,124 | 4,166 |
| Conservative | Cilostazol only | 30.9% | 16.9% | 146 | 194 |
| Conservative | Combination | 30.9% | 16.7% | 142 | 189 |

*Note. Values use equal allocation within each separate active-versus-control calculation, 80% power, and a two-sided normal approximation for two independent proportions. Alpha 0.0167 is a Bonferroni sensitivity analysis for three active-versus-control comparisons. Each row gives the common per-group size required to power that specific contrast. An equally allocated four-arm design would ordinarily select a common per-group target based on the most demanding prespecified primary contrast and its operating characteristics; total enrollment would then be four times that target. These calculations are illustrative and do not power the factorial interaction or account for adaptive monitoring, missingness, or a final trial-specific multiplicity strategy.*

**Supplementary Table 5.** Sensitivity to neutral and diffuse neutral albumin priors.

| **Setting** | **Albumin RR prior, median (95% interval)** | **Albumin ARR, median pp (90% interval); Pr ARR >0** | **Combination ARR and incremental albumin effect, median pp (90% interval)** | **Probability of lowest-risk class: cilostazol; combination** |
| --- | --- | --- | --- | --- |
| Base case | 0.70 (0.40-1.20) | 9.1 (-3.3 to 18.1); 89.8% | 21.7 (12.4 to 29.9); 3.5 (-3.0 to 8.9) | 15.1%; 84.7% |
| Neutral albumin | 1.00 (0.67-1.50) | 0.0 (-12.6 to 9.0); 50.0% | 18.0 (7.1 to 26.7); 0.0 (-8.0 to 5.1) | 50.0%; 49.9% |
| Diffuse neutral albumin | 1.00 (0.50-2.00) | 0.0 (-24.7 to 13.8); 50.0% | 18.0 (3.3 to 27.6); 0.0 (-12.6 to 6.6) | 50.0%; 49.9% |

*Note. Cilostazol and interaction priors were held at their base-case values. Incremental albumin effect is the ARR for albumin added to cilostazol. Values are prior-predictive summaries, not observed treatment effects.*

*Abbreviations: ARR, absolute risk reduction; pp, percentage points; Pr, probability; RR, relative risk.*

**Supplementary Table 6.** Sensitivity to correlation among log-relative-risk priors.

| **Prior-dependence setting** | **Correlation structure** | **Combination ARR, median pp (90% interval)** | **Incremental albumin effect, median pp (90% interval)** | **Pr combination lowest-risk class** |
| --- | --- | --- | --- | --- |
| Independent | All pairwise ρ = 0 | 21.7 (12.4 to 29.9) | 3.5 (-3.0 to 8.9) | 84.7% |
| Positive component correlation | ρ(A,C) = +0.50; interaction independent | 21.6 (10.3 to 30.3) | 3.4 (-3.5 to 7.7) | 84.8% |
| Negative component correlation | ρ(A,C) = -0.50; interaction independent | 21.8 (14.3 to 29.4) | 3.5 (-2.5 to 10.1) | 84.5% |
| Positive joint correlation | All pairwise ρ = +0.50 | 21.5 (7.0 to 30.8) | 3.4 (-6.4 to 7.6) | 80.0% |

*Note. Marginal base-case priors were unchanged. Correlations were imposed on log(RR_A), log(RR_C), and log(RR_AC). These prespecified structures assess sensitivity to epistemic dependence and are not estimated from observed data.*

*Abbreviations: A, albumin; ARR, absolute risk reduction; C, cilostazol; pp, percentage points; Pr, probability; ρ, correlation coefficient.*

**Supplementary Note 1. Control-risk assumption.**

The CATS protocol specifies an expected control CDE-d-DCI risk of 31% based on values derived from preliminary published data corresponding to references 9, 30, and 31 of the main manuscript. This value is treated as a planning input rather than observed CATS trial data. Supplementary Table 3 evaluates alternative control-risk means and effective sample sizes.

**Supplementary Note 2. Dose and duration pooling.**

Dose-specific evidence remains important, but the 300 mg/day versus 100 to 200 mg/day cilostazol comparison was observational and was not used to assign dose-specific priors. ALISAH varied daily albumin dose, whereas CATS varies treatment duration at 1.25 g/kg/day; therefore, the available ALISAH data do not provide a direct basis for duration-specific albumin priors. These limitations support retaining the full factorial dose and duration structure in future CATS analyses while using class-level estimates only for planning-level interpretability.

**Supplementary Note 3. Albumin prior calibration and skeptical alternatives.**

The albumin prior was not obtained through formal multi-expert elicitation. ALISAH tier-specific DCI proportions of 20%, 15%, and 14%, compared descriptively with the 31% planning control risk, correspond to crude cross-study risk ratios of approximately 0.65, 0.48, and 0.45. A representative ratio of 0.48 was discounted halfway toward the null on the log-relative-risk scale, yielding exp{0.5 x log(0.48)} = 0.69, rounded to 0.70. The 95% interval 0.40 to 1.20 was selected to include no effect and plausible harm. Because the anchor is uncontrolled and the discount is judgmental, Supplementary Table 5 repeats the analysis using neutral and diffuse neutral albumin priors centered at RR 1.00.

**Supplementary Note 4. Prior dependence and alternative Bayesian formulations.**

The interaction parameter permits departure from multiplicative component effects and therefore addresses biological overlap separately from correlation among parameter uncertainties. The base case samples the three log-relative-risk parameters independently because their covariance is not identifiable from the available aggregate evidence. Supplementary Table 6 imposes moderate prespecified correlations as stress tests. Future analyses could use a multivariate prior with elicited covariance, robust-mixture priors, meta-analytic-predictive or commensurate/power priors that discount nonexchangeable external evidence, or hierarchical partial pooling across albumin duration and cilostazol dose cells.

**Supplementary Figure 1.** Base-case prior-predictive absolute risk reductions for NINDS CDE-defined DCI.


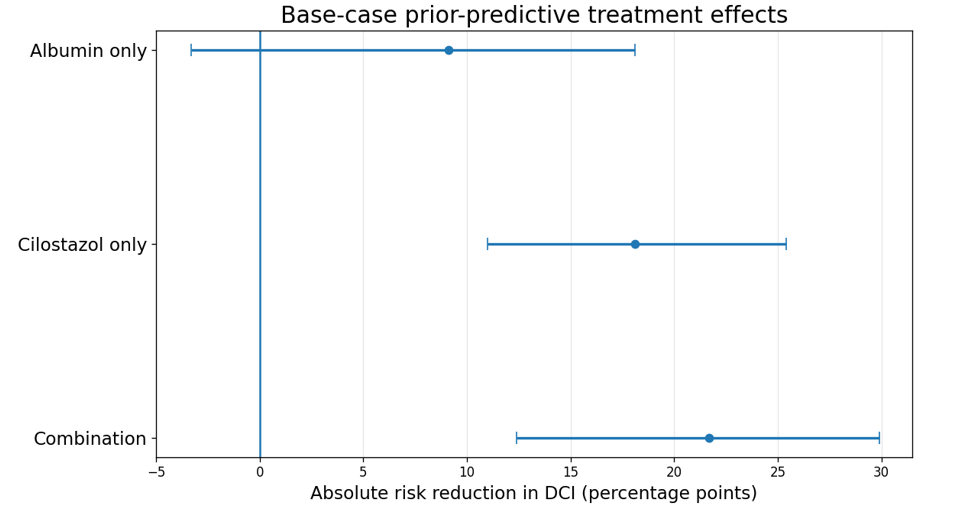


*Points are medians; horizontal bars are 90% prior-predictive intervals. Estimates are simulation-based projections, not observed results.*

**Supplementary Figure 2.** Sensitivity of treatment-effect projections to prior assumptions.


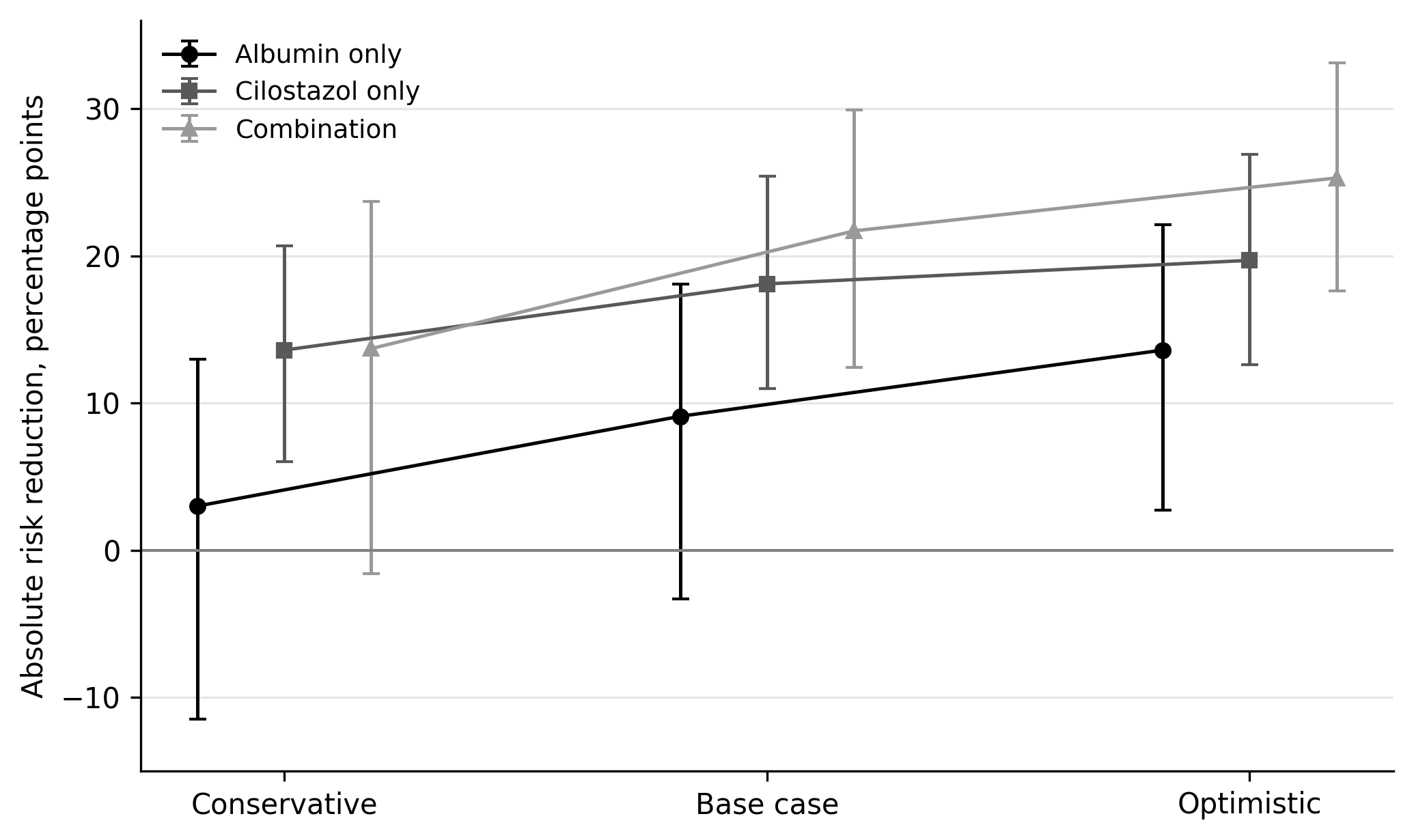


*Median absolute risk reductions and 90% prior-predictive intervals are shown across*

*conservative, base-case, and optimistic scenarios.*

**Supplementary Appendix 2. Reproducible prior-predictive and component-effect analysis script**

**Purpose.** This appendix provides a reproducible base R script for the prior-predictive treatment-effect simulations, sensitivity analyses, combination interaction summaries, external-consistency table, control-prior sensitivity analyses, weaker albumin-prior sensitivity analyses, prior-dependence sensitivity analyses, and illustrative sample-size calculations for albumin, cilostazol, and combination therapy after aneurysmal subarachnoid hemorrhage. The script generates CSV outputs corresponding to the manuscript and supplementary tables.

**Software requirements.** Base R only; no additional packages are required. Recommended command: Rscript CATS_Reproducible_Prior_Predictive_Analysis_Supplement.R

**Interpretation.** The output represents Monte Carlo prior-predictive summaries based on protocol assumptions and published aggregate evidence. These are not individual-level CATS trial results or posterior estimates of clinical effectiveness. Small changes in the final decimal may occur if the seed, number of simulations, or R random-number generator changes.

# R script

# Supplementary Appendix 2

# Reproducible prior-predictive and component-effect analysis script

# CATS Bayesian simulation and component-effect manuscript

#

# Software: Base R only. No additional packages are required.

# Recommended run:

# Rscript CATS_Reproducible_Prior_Predictive_Analysis_Supplement.R

#

# This script reproduces the prior-predictive treatment-effect estimates,

# sensitivity analyses, and combination interaction summaries for albumin,

# cilostazol, and combination therapy after aneurysmal subarachnoid hemorrhage.

# The analysis uses protocol assumptions and published aggregate evidence. It is

# not an analysis of individual-level CATS trial outcomes.

set.seed(20260508)

NSIM <- 300000

CONTROL_ALPHA <- 31

CONTROL_BETA <- 69

OUTDIR <- getwd()

# -----------------------------------------------------------------------------

# Helper functions

# -----------------------------------------------------------------------------

draw_rr <- function(n, median_rr, lo95, hi95) {

# Draw relative risks from a log-normal distribution parameterized by a median

# and an approximate central 95% prior interval.

sdlog <- (log(hi95) - log(lo95)) / (2 * qnorm(0.975))

rlnorm(n = n, meanlog = log(median_rr), sdlog = sdlog)

}

fmt_pct <- function(x, digits = 1) {

paste0(formatC(100 * x, format = "f", digits = digits), "%")

}

fmt_pp <- function(x, digits = 1) {

formatC(100 * x, format = "f", digits = digits)

}

fmt_interval_pct <- function(center, lo, hi, digits = 1) {

paste0(fmt_pct(center, digits), " (", fmt_pp(lo, digits), " to ",

fmt_pp(hi, digits), ")")

}

fmt_interval_pp <- function(center, lo, hi, digits = 1) {

paste0(fmt_pp(center, digits), " (", fmt_pp(lo, digits), " to ",

fmt_pp(hi, digits), ")")

}

fmt_interval_rr <- function(center, lo, hi, digits = 2) {

paste0(formatC(center, format = "f", digits = digits), " (",

formatC(lo, format = "f", digits = digits), "-",

formatC(hi, format = "f", digits = digits), ")")

}

summarize_treatment <- function(p0, pt) {

arr <- p0 - pt

rr <- pt / p0

data.frame(

risk_median = median(pt),

risk_q05 = as.numeric(quantile(pt, 0.05)),

risk_q95 = as.numeric(quantile(pt, 0.95)),

arr_median = median(arr),

arr_q05 = as.numeric(quantile(arr, 0.05)),

arr_q95 = as.numeric(quantile(arr, 0.95)),

rr_median = median(rr),

rr_q05 = as.numeric(quantile(rr, 0.05)),

rr_q95 = as.numeric(quantile(rr, 0.95)),

pr_arr_gt_5pp = mean(arr > 0.05),

pr_arr_gt_10pp = mean(arr > 0.10),

pr_arr_gt_0 = mean(arr > 0.00)

)

}

simulate_scenario <- function(label, albumin_prior, cilostazol_prior,

interaction_prior, nsim = NSIM) {

# Draw baseline control risk.

p0 <- rbeta(nsim, CONTROL_ALPHA, CONTROL_BETA)

# Draw component relative risks.

rr_albumin <- draw_rr(

nsim,

median_rr = albumin_prior["median"],

lo95 = albumin_prior["lo95"],

hi95 = albumin_prior["hi95"]

)

rr_cilostazol <- draw_rr(

nsim,

median_rr = cilostazol_prior["median"],

lo95 = cilostazol_prior["lo95"],

hi95 = cilostazol_prior["hi95"]

)

rr_interaction <- draw_rr(

nsim,

median_rr = interaction_prior["median"],

lo95 = interaction_prior["lo95"],

hi95 = interaction_prior["hi95"]

)

# Risks under each treatment class.

p_albumin <- p0 * rr_albumin

p_cilostazol <- p0 * rr_cilostazol

p_combination <- p0 * rr_albumin * rr_cilostazol * rr_interaction

# Keep probabilities within [0, 1]. Truncation is uncommon under these priors.

p_albumin <- pmin(pmax(p_albumin, 0), 1)

p_cilostazol <- pmin(pmax(p_cilostazol, 0), 1)

p_combination <- pmin(pmax(p_combination, 0), 1)

arr_albumin <- p0 - p_albumin

arr_cilostazol <- p0 - p_cilostazol

arr_combination <- p0 - p_combination

additive_interaction <- arr_combination - arr_albumin - arr_cilostazol

risks <- cbind(

albumin = p_albumin,

cilostazol = p_cilostazol,

combination = p_combination

)

best_index <- max.col(-risks, ties.method = "random")

list(

label = label,

p0 = p0,

risks = risks,

arr = cbind(

albumin = arr_albumin,

cilostazol = arr_cilostazol,

combination = arr_combination

),

treatment_summary = rbind(

albumin = summarize_treatment(p0, p_albumin),

cilostazol = summarize_treatment(p0, p_cilostazol),

combination = summarize_treatment(p0, p_combination)

),

additive_interaction = additive_interaction,

pr_supra_additive = mean(additive_interaction > 0),

pr_best = c(

albumin = mean(best_index == 1),

cilostazol = mean(best_index == 2),

combination = mean(best_index == 3)

)

)

}

# -----------------------------------------------------------------------------

# Prior specification

# -----------------------------------------------------------------------------

priors <- list(

conservative = list(

albumin = c(median = 0.90, lo95 = 0.55, hi95 = 1.50),

cilostazol = c(median = 0.55, lo95 = 0.35, hi95 = 0.80),

interaction = c(median = 1.10, lo95 = 0.75, hi95 = 1.60)

),

base_case = list(

albumin = c(median = 0.70, lo95 = 0.40, hi95 = 1.20),

cilostazol = c(median = 0.40, lo95 = 0.24, hi95 = 0.67),

interaction = c(median = 1.00, lo95 = 0.67, hi95 = 1.50)

),

optimistic = list(

albumin = c(median = 0.55, lo95 = 0.30, hi95 = 1.00),

cilostazol = c(median = 0.35, lo95 = 0.20, hi95 = 0.60),

interaction = c(median = 0.85, lo95 = 0.50, hi95 = 1.35)

)

)

# -----------------------------------------------------------------------------

# Run simulations

# -----------------------------------------------------------------------------

scenarios <- list(

conservative = simulate_scenario(

"Conservative",

priors$conservative$albumin,

priors$conservative$cilostazol,

priors$conservative$interaction

),

base_case = simulate_scenario(

"Base case",

priors$base_case$albumin,

priors$base_case$cilostazol,

priors$base_case$interaction

),

optimistic = simulate_scenario(

"Optimistic",

priors$optimistic$albumin,

priors$optimistic$cilostazol,

priors$optimistic$interaction

)

)

# -----------------------------------------------------------------------------

# Intermediate output A. Base-case prior-predictive treatment-effect estimates

# -----------------------------------------------------------------------------

base <- scenarios$base_case$treatment_summary

base$treatment <- c("Albumin only", "Cilostazol only", "Combination")

table3 <- data.frame(

Treatment_class = base$treatment,

Projected_risk_median_90_interval = mapply(

fmt_interval_pct,

base$risk_median, base$risk_q05, base$risk_q95

),

ARR_median_pp_90_interval = mapply(

fmt_interval_pp,

base$arr_median, base$arr_q05, base$arr_q95

),

RR_median_90_interval = mapply(

fmt_interval_rr,

base$rr_median, base$rr_q05, base$rr_q95

),

Pr_ARR_gt_5pp = fmt_pct(base$pr_arr_gt_5pp),

Pr_ARR_gt_10pp = fmt_pct(base$pr_arr_gt_10pp),

Pr_ARR_gt_0 = fmt_pct(base$pr_arr_gt_0),

row.names = NULL

)

# -----------------------------------------------------------------------------

# Intermediate output B. Prior-sensitivity analysis for absolute risk reduction

# -----------------------------------------------------------------------------

arr_summary <- function(x, treatment) {

arr <- x$arr[, treatment]

fmt_interval_pp(

median(arr),

as.numeric(quantile(arr, 0.05)),

as.numeric(quantile(arr, 0.95))

)

}

table4 <- data.frame(

Scenario = sapply(scenarios, function(x) x$label),

Albumin_only_ARR_pp = sapply(scenarios, arr_summary, treatment = "albumin"),

Cilostazol_only_ARR_pp = sapply(scenarios, arr_summary, treatment = "cilostazol"),

Combination_ARR_pp = sapply(scenarios, arr_summary, treatment = "combination"),

row.names = NULL

)

# -----------------------------------------------------------------------------

# Intermediate output C. Combination interaction and probability of lowest-risk active-treatment class

# -----------------------------------------------------------------------------

interaction_summary <- function(x) {

z <- x$additive_interaction

fmt_interval_pp(

median(z),

as.numeric(quantile(z, 0.05)),

as.numeric(quantile(z, 0.95))

)

}

table5 <- data.frame(

Scenario = sapply(scenarios, function(x) x$label),

Additive_interaction_pp = sapply(scenarios, interaction_summary),

Pr_supra_additive = fmt_pct(sapply(scenarios, function(x) x$pr_supra_additive)),

Pr_albumin_best = fmt_pct(sapply(scenarios, function(x) x$pr_best["albumin"])),

Pr_cilostazol_best = fmt_pct(sapply(scenarios, function(x) x$pr_best["cilostazol"])),

Pr_combination_best = fmt_pct(sapply(scenarios, function(x) x$pr_best["combination"])),

row.names = NULL

)

# -----------------------------------------------------------------------------

# Consolidated table combining intermediate outputs A, B, and C

# -----------------------------------------------------------------------------

combined_rows <- list()

for (i in seq_len(nrow(table3))) {

combined_rows[[length(combined_rows) + 1]] <- data.frame(

Panel = "A. Base-case treatment effect",

Scenario = "Base case",

Treatment_or_quantity = table3$Treatment_class[i],

Projected_risk = table3$Projected_risk_median_90_interval[i],

ARR = table3$ARR_median_pp_90_interval[i],

RR = table3$RR_median_90_interval[i],

Pr_ARR_gt_5pp = table3$Pr_ARR_gt_5pp[i],

Pr_ARR_gt_10pp = table3$Pr_ARR_gt_10pp[i],

Pr_ARR_gt_0 = table3$Pr_ARR_gt_0[i],

Additive_interaction_pp = "",

Pr_supra_additive = "",

Pr_albumin_best = "",

Pr_cilostazol_best = "",

Pr_combination_best = ""

)

}

for (i in seq_len(nrow(table4))) {

for (j in c("Albumin only", "Cilostazol only", "Combination")) {

arr_value <- if (j == "Albumin only") {

table4$Albumin_only_ARR_pp[i]

} else if (j == "Cilostazol only") {

table4$Cilostazol_only_ARR_pp[i]

} else {

table4$Combination_ARR_pp[i]

}

combined_rows[[length(combined_rows) + 1]] <- data.frame(

Panel = "B. Prior-sensitivity absolute risk reduction",

Scenario = table4$Scenario[i],

Treatment_or_quantity = j,

Projected_risk = "",

ARR = arr_value,

RR = "",

Pr_ARR_gt_5pp = "",

Pr_ARR_gt_10pp = "",

Pr_ARR_gt_0 = "",

Additive_interaction_pp = "",

Pr_supra_additive = "",

Pr_albumin_best = "",

Pr_cilostazol_best = "",

Pr_combination_best = ""

)

}

}

for (i in seq_len(nrow(table5))) {

combined_rows[[length(combined_rows) + 1]] <- data.frame(

Panel = "C. Combination interaction and lowest-risk active-treatment class",

Scenario = table5$Scenario[i],

Treatment_or_quantity = "Combination interaction / lowest-risk active-treatment class",

Projected_risk = "",

ARR = "",

RR = "",

Pr_ARR_gt_5pp = "",

Pr_ARR_gt_10pp = "",

Pr_ARR_gt_0 = "",

Additive_interaction_pp = table5$Additive_interaction_pp[i],

Pr_supra_additive = table5$Pr_supra_additive[i],

Pr_albumin_best = table5$Pr_albumin_best[i],

Pr_cilostazol_best = table5$Pr_cilostazol_best[i],

Pr_combination_best = table5$Pr_combination_best[i]

)

}

combined_table <- do.call(rbind, combined_rows)

# -----------------------------------------------------------------------------

# Reviewer-requested external-consistency, control-prior sensitivity, and

# illustrative sample-size analyses

# -----------------------------------------------------------------------------

external_consistency <- data.frame(

Evidence_domain = c("Control-risk context", "Cilostazol", "Albumin", "Combination therapy"),

Published_aggregate_data = c(

"New cerebral infarction: 26.6% across pooled control groups from randomized and observational studies (reference 25)",

"New cerebral infarction: 10.1% with cilostazol versus 26.6% control across randomized and observational studies; randomized-trial RR 0.40 (95% CI 0.24-0.67) (reference 25)",

"ALISAH DCI: 20%, 15%, and 14%; cerebral infarction: 45%, 27%, and 25% among patients with follow-up CT (references 28 and 29)",

"No directly comparable randomized human data"

),

Base_case_projection = c(

"Control risk 30.9% (90% interval 23.7-38.8)",

"Risk 12.3% versus 30.9%; median RR 0.40 (90% interval 0.26-0.62)",

"Albumin risk 21.5% (90% interval 12.7-36.1)",

"Combination risk 8.6% (90% interval 4.0-18.3)"

),

Interpretation = c(

"Contextual consistency; 31% is the CATS planning value supported by published data",

"Consistency expected because the randomized-trial RR informed the prior; not independent validation",

"Descriptive comparison only because ALISAH was uncontrolled and endpoints differed",

"External validation is not currently possible"

),

stringsAsFactors = FALSE

)

simulate_control_prior <- function(mean_risk, ess, nsim = NSIM) {

set.seed(20260508)

p0 <- rbeta(nsim, mean_risk * ess, (1 - mean_risk) * ess)

rr_albumin <- draw_rr(

nsim,

priors$base_case$albumin["median"],

priors$base_case$albumin["lo95"],

priors$base_case$albumin["hi95"]

)

rr_cilostazol <- draw_rr(

nsim,

priors$base_case$cilostazol["median"],

priors$base_case$cilostazol["lo95"],

priors$base_case$cilostazol["hi95"]

)

rr_interaction <- draw_rr(

nsim,

priors$base_case$interaction["median"],

priors$base_case$interaction["lo95"],

priors$base_case$interaction["hi95"]

)

p_albumin <- pmin(pmax(p0 * rr_albumin, 0), 1)

p_cilostazol <- pmin(pmax(p0 * rr_cilostazol, 0), 1)

p_combination <- pmin(pmax(p0 * rr_albumin * rr_cilostazol * rr_interaction, 0), 1)

list(

p0 = p0,

albumin = p_albumin,

cilostazol = p_cilostazol,

combination = p_combination

)

}

format_control_sensitivity <- function(mean_risk, ess) {

if (isTRUE(all.equal(mean_risk, 0.31)) && ess == 100) {

x <- list(

p0 = scenarios$base_case$p0,

albumin = scenarios$base_case$risks[, "albumin"],

cilostazol = scenarios$base_case$risks[, "cilostazol"],

combination = scenarios$base_case$risks[, "combination"]

)

} else {

x <- simulate_control_prior(mean_risk, ess)

}

data.frame(

Control_prior_setting = paste0(

"Mean ", formatC(100 * mean_risk, format = "f", digits = 1),

"%; ESS ", ess

),

Control_risk = fmt_interval_pct(

median(x$p0), quantile(x$p0, 0.05), quantile(x$p0, 0.95)

),

Albumin_ARR_pp = fmt_interval_pp(

median(x$p0 - x$albumin),

quantile(x$p0 - x$albumin, 0.05),

quantile(x$p0 - x$albumin, 0.95)

),

Cilostazol_ARR_pp = fmt_interval_pp(

median(x$p0 - x$cilostazol),

quantile(x$p0 - x$cilostazol, 0.05),

quantile(x$p0 - x$cilostazol, 0.95)

),

Combination_ARR_pp = fmt_interval_pp(

median(x$p0 - x$combination),

quantile(x$p0 - x$combination, 0.05),

quantile(x$p0 - x$combination, 0.95)

),

row.names = NULL

)

}

control_mean_sensitivity <- do.call(

rbind,

lapply(c(0.266, 0.31, 0.35), function(m) format_control_sensitivity(m, 100))

)

control_ess_sensitivity <- do.call(

rbind,

lapply(c(25, 50, 100, 200), function(e) format_control_sensitivity(0.31, e))

)

control_prior_sensitivity <- rbind(control_mean_sensitivity, control_ess_sensitivity)

required_n_two_proportions <- function(p_control, p_active, alpha = 0.05, power = 0.80) {

z_alpha <- qnorm(1 - alpha / 2)

z_power <- qnorm(power)

p_bar <- (p_control + p_active) / 2

ceiling(

((z_alpha * sqrt(2 * p_bar * (1 - p_bar)) +

z_power * sqrt(p_control * (1 - p_control) + p_active * (1 - p_active)))^2) /

(p_control - p_active)^2

)

}

sample_size_rows <- list()

for (scenario_name in c("base_case", "conservative")) {

x <- scenarios[[scenario_name]]

p_control <- median(x$p0)

for (treatment in c("albumin", "cilostazol", "combination")) {

p_active <- median(x$risks[, treatment])

sample_size_rows[[length(sample_size_rows) + 1]] <- data.frame(

Scenario = x$label,

Comparison = paste0(treatment, " versus control"),

Control_risk = fmt_pct(p_control),

Active_risk = fmt_pct(p_active),

N_per_group_alpha_0_05 = required_n_two_proportions(

p_control, p_active, 0.05, 0.80

),

N_per_group_alpha_0_0167 = required_n_two_proportions(

p_control, p_active, 0.05 / 3, 0.80

),

row.names = NULL

)

}

}

illustrative_sample_size <- do.call(rbind, sample_size_rows)

# -----------------------------------------------------------------------------

# Reviewer 2 sensitivity analyses: skeptical albumin priors and prior dependence

# -----------------------------------------------------------------------------

summarize_reviewer2_scenario <- function(label, albumin_prior) {

set.seed(20260508)

x <- simulate_scenario(

label,

albumin_prior,

priors$base_case$cilostazol,

priors$base_case$interaction

)

albumin_arr <- x$arr[, "albumin"]

combination_arr <- x$arr[, "combination"]

incremental_albumin <- x$risks[, "cilostazol"] - x$risks[, "combination"]

data.frame(

Setting = label,

Albumin_prior = paste0(

albumin_prior["median"], " (", albumin_prior["lo95"], "-",

albumin_prior["hi95"], ")"

),

Albumin_ARR_pp = fmt_interval_pp(

median(albumin_arr), quantile(albumin_arr, 0.05), quantile(albumin_arr, 0.95)

),

Pr_albumin_ARR_gt_0 = fmt_pct(mean(albumin_arr > 0)),

Combination_ARR_pp = fmt_interval_pp(

median(combination_arr), quantile(combination_arr, 0.05),

quantile(combination_arr, 0.95)

),

Incremental_albumin_on_cilostazol_pp = fmt_interval_pp(

median(incremental_albumin), quantile(incremental_albumin, 0.05),

quantile(incremental_albumin, 0.95)

),

Pr_cilostazol_lowest_risk = fmt_pct(x$pr_best["cilostazol"]),

Pr_combination_lowest_risk = fmt_pct(x$pr_best["combination"]),

row.names = NULL

)

}

albumin_prior_sensitivity <- rbind(

summarize_reviewer2_scenario("Base case", priors$base_case$albumin),

summarize_reviewer2_scenario(

"Neutral albumin",

c(median = 1.00, lo95 = 0.67, hi95 = 1.50)

),

summarize_reviewer2_scenario(

"Diffuse neutral albumin",

c(median = 1.00, lo95 = 0.50, hi95 = 2.00)

)

)

simulate_correlated_priors <- function(label, correlation_matrix, nsim = NSIM) {

set.seed(20260508)

p0 <- rbeta(nsim, CONTROL_ALPHA, CONTROL_BETA)

marginal_priors <- list(

priors$base_case$albumin,

priors$base_case$cilostazol,

priors$base_case$interaction

)

meanlog <- sapply(marginal_priors, function(x) log(x["median"]))

sdlog <- sapply(

marginal_priors,

function(x) (log(x["hi95"]) - log(x["lo95"])) / (2 * qnorm(0.975))

)

z <- matrix(rnorm(nsim * 3), ncol = 3)

correlated_z <- z %*% chol(correlation_matrix)

log_rr <- sweep(correlated_z, 2, sdlog, "*")

log_rr <- sweep(log_rr, 2, meanlog, "+")

rr <- exp(log_rr)

p_albumin <- pmin(pmax(p0 * rr[, 1], 0), 1)

p_cilostazol <- pmin(pmax(p0 * rr[, 2], 0), 1)

p_combination <- pmin(pmax(p0 * rr[, 1] * rr[, 2] * rr[, 3], 0), 1)

combination_arr <- p0 - p_combination

incremental_albumin <- p_cilostazol - p_combination

# Ranking is restricted to the three active-treatment classes; control is excluded.

best_index <- max.col(-cbind(p_albumin, p_cilostazol, p_combination), ties.method = "random")

data.frame(

Setting = label,

Combination_ARR_pp = fmt_interval_pp(

median(combination_arr), quantile(combination_arr, 0.05),

quantile(combination_arr, 0.95)

),

Incremental_albumin_on_cilostazol_pp = fmt_interval_pp(

median(incremental_albumin), quantile(incremental_albumin, 0.05),

quantile(incremental_albumin, 0.95)

),

Pr_combination_lowest_risk = fmt_pct(mean(best_index == 3)),

row.names = NULL

)

}

R_independent <- diag(3)

R_positive_components <- matrix(

c(1, 0.50, 0, 0.50, 1, 0, 0, 0, 1), nrow = 3, byrow = TRUE

)

R_negative_components <- matrix(

c(1, -0.50, 0, -0.50, 1, 0, 0, 0, 1), nrow = 3, byrow = TRUE

)

R_positive_joint <- matrix(0.50, nrow = 3, ncol = 3)

diag(R_positive_joint) <- 1

prior_dependence_sensitivity <- rbind(

simulate_correlated_priors("Independent", R_independent),

simulate_correlated_priors("Positive component correlation", R_positive_components),

simulate_correlated_priors("Negative component correlation", R_negative_components),

simulate_correlated_priors("Positive joint correlation", R_positive_joint)

)

# -----------------------------------------------------------------------------

# Print and write outputs

# -----------------------------------------------------------------------------

cat("\nIntermediate output A. Base-case prior-predictive treatment-effect estimates\n")

print(table3, row.names = FALSE)

cat("\nIntermediate output B. Prior-sensitivity analysis for absolute risk reduction\n")

print(table4, row.names = FALSE)

cat("\nIntermediate output C. Combination interaction and probability of lowest-risk active-treatment class\n")

print(table5, row.names = FALSE)

cat("\nConsolidated table combining intermediate outputs A, B, and C\n")

print(combined_table, row.names = FALSE)

cat("\nSupplementary Table 2. External consistency\n")

print(external_consistency, row.names = FALSE)

cat("\nSupplementary Table 3. Control-prior sensitivity\n")

print(control_prior_sensitivity, row.names = FALSE)

cat("\nSupplementary Table 4. Illustrative sample sizes\n")

print(illustrative_sample_size, row.names = FALSE)

cat("\nSupplementary Table 5. Sensitivity to neutral and diffuse neutral albumin priors\n")

print(albumin_prior_sensitivity, row.names = FALSE)

cat("\nSupplementary Table 6. Sensitivity to correlation among log-relative-risk priors\n")

print(prior_dependence_sensitivity, row.names = FALSE)

write.csv(table3, file.path(OUTDIR, "cats_table3_basecase_prior_predictive.csv"), row.names = FALSE)

write.csv(table4, file.path(OUTDIR, "cats_table4_sensitivity_arr.csv"), row.names = FALSE)

write.csv(table5, file.path(OUTDIR, "cats_table5_interaction_best.csv"), row.names = FALSE)

write.csv(combined_table, file.path(OUTDIR, "cats_consolidated_tables_3_to_5.csv"), row.names = FALSE)

write.csv(external_consistency, file.path(OUTDIR, "cats_supp_table2_external_consistency.csv"), row.names = FALSE)

write.csv(control_prior_sensitivity, file.path(OUTDIR, "cats_supp_table3_control_prior_sensitivity.csv"), row.names = FALSE)

write.csv(illustrative_sample_size, file.path(OUTDIR, "cats_supp_table4_illustrative_sample_size.csv"), row.names = FALSE)

write.csv(albumin_prior_sensitivity, file.path(OUTDIR, "cats_supp_table5_albumin_prior_sensitivity.csv"), row.names = FALSE)

write.csv(prior_dependence_sensitivity, file.path(OUTDIR, "cats_supp_table6_prior_dependence_sensitivity.csv"), row.names = FALSE)

cat("\nCSV files written to: ", OUTDIR, "\n", sep = "")

cat("Note: values are Monte Carlo prior-predictive summaries. They may differ in\n")

cat("the final decimal if the seed, NSIM, or R random-number generator changes.\n")

**Supplementary Appendix 3. Generative AI prompts**

**Initial prompt:**
Refine the Methods section describing the Bayesian prior-predictive Monte Carlo simulation model, prior distributions, treatment-class risk estimates, absolute risk reduction calculations, and factorial component-effect decomposition for albumin, cilostazol, and combination therapy after aneurysmal subarachnoid hemorrhage.

**Final prompt:**
Review the manuscript for grammar, clarity, consistency of terminology, abbreviation use, and word count compliance, while preserving the scientific meaning and author-provided data.
